# Development of a Complex Biologically Active Supplement for Immunomodulation

**DOI:** 10.3390/foods14234072

**Published:** 2025-11-27

**Authors:** Gulzhan Zhumaliyeva, Urishbay Chomanov, Gulmira Kenenbay, Assiya Shoman, Ainel Baizakova, Shynar Akzholtayeva

**Affiliations:** Almaty Branch of LLP “Kazakh Research Institute of Processing and Food Industry”, Serkebayev Avenue, 62, Almaty 050060, Kazakhstan; u.chomanov@bk.ru (U.C.); g.kenenbay@bk.ru (G.K.); shoman_a@mail.ru (A.S.);

**Keywords:** Jerusalem artichoke, sprouted oats, sprouted barley, liquorice root, biologically active additives

## Abstract

In light of the increasing prevalence of metabolic disorders and immune-deficiency conditions, the development of complex plant-based biologically active supplements (BAS) represents a pressing challenge in modern food science. The aim of this study was to develop an immunomodulatory BAS using Jerusalem artichoke, sprouted oats, sprouted barley, and licorice root. Physicochemical, organoleptic, and microbiological analyses of raw materials and experimental samples were performed. It was established that sprouted grains are characterized by increased protein content (oats—12.64%, barley—11.87%) and elevated levels of amino acids (lysine—1.42% in sprouted barley). Jerusalem artichoke demonstrated high levels of dietary fiber (24.65%) and vitamin C (31.95 mg/100 g), while licorice root contained significant amounts of glycyrrhizic acid and vitamin B_2_ (0.77 mg/100 g). The combination of Jerusalem artichoke, sprouted grains, and licorice root forms a solid foundation for the development of a complex BAS capable of normalizing metabolism and supporting the immune system, particularly in individuals with diabetes mellitus. This approach aligns with current trends in functional nutrition and contributes to import substitution and the advancement of Kazakhstan’s agro-industrial sector. Four BAS formulations were evaluated, and Sample 4 (Jerusalem artichoke—60 g, sprouted oats—12.5 g, sprouted barley—12.5 g, licorice root—15 g) was identified as optimal due to its balanced composition and high technological performance. It demonstrated good flowability (angle of repose—34°), satisfactory water-holding capacity (0.701 g/g), and the highest stability in organoleptic characteristics. The protein content of this sample was 11.97%, fiber—9.24%, and vitamin E—57.75 mg/100 g. The results confirm that the developed BAS is a valuable source of dietary fiber, amino acids, vitamins, and minerals, providing a pronounced synergistic immunomodulatory effect. The practical significance of the study lies in the potential application of the developed composition in the production of functional foods aimed at metabolic correction and diabetes prevention.

## 1. Introduction

Preserving and strengthening human health is one of the key priorities of any civilized state. Long before the emergence of nutrition science, philosophers and later physicians recognized the direct link between diet and health. Today, it is well established that health care systems contribute only 8–12% to overall population health, whereas socio-economic factors, including dietary patterns, account for 52–55% [1].

The exchange of macro- and microelements in the human body, along with the disorders associated with it, remain among the pressing challenges of modern nutrition science. This issue is inherently interdisciplinary, as maintaining elemental balance cannot be addressed solely from a clinical or biochemical perspective.

Despite the availability of international and national dietary guidelines, population adherence to them remains extremely low. Analysis of multiple UK data sources—including the National Diet and Nutrition Survey (NDNS)—revealed that only 0.078% of individuals met all nine recommendations of the British Eatwell Guide. Approximately 44% adhered to only 3–4 recommendations, and about 30% met five or more. Higher adherence correlated with a reduced risk of all-cause mortality and a lower dietary carbon footprint [2]. This highlights a substantial discrepancy between recommended dietary standards and actual eating behaviors.

Similar trends were observed in a large UK Biobank cohort. Kebbe et al. (2021) [3] reported that only 9.5% of participants met three to four key WHO recommendations on fruit and vegetable intake, dietary fiber, saturated fats, and free sugars. Nearly one-third did not meet a single recommendation, and fewer than 0.1% met all nine. The study emphasizes the very low adherence to healthy dietary practices in real-world conditions.

A systematic review by Leme et al. (2021) [4], covering countries with varying income levels, confirmed that inadequate adherence to dietary guidelines is a global issue. In many regions, up to 40% of the population did not meet any recommendations, while compliance with fruit and vegetable intake ranged from 7% to 67% depending on the country and assessment method.

Elemental balance in the human body fluctuates due to multiple factors, primarily intake and excretion. Their interaction results in variations of up to 20% throughout the year [2,3].

Dietary supplements have therefore become increasingly common. Following the COVID-19 pandemic, their demand rose sharply. They are used for prevention, during illness, and throughout recovery. As biologically active food supplements, they help compensate for potential nutrient deficiencies. Chronic deficits in vitamins, trace elements, and macro- and micronutrients disrupt homeostasis and impair the functioning of organs and systems. Moreover, some essential substances cannot be synthesized by the body, making external intake necessary. Regular use of dietary supplements may reduce the risk of deficiency-related conditions by up to 80% [4,5].

The goals of dietary supplements include disease prevention, slowing the aging process, increasing longevity, supporting normal physiological functioning, and improving overall health.

Ideally, all necessary nutrients should be obtained from food—vegetables, fruits, meat, fish, and dairy products. However, achieving a varied and balanced diet remains rare: fewer than 1% of adults meet these standards [6,7,8]. Therefore, it is reasonable to conclude that dietary supplements are needed by the vast majority of the population, with the exception of infants.

Dietary supplements can be a sensitive topic, evoking a wide range of opinions and viewpoints. While some observers argue that these products should be treated similarly to conventional medicines and food products, others believe a more personalized approach is necessary.

At the national level, countries seek to adjust the nutrition of their populations according to modern requirements. For this reason, at the initiative of the World Health Organization, food supplements have been developed and introduced worldwide, rapidly becoming widespread. Such supplements include natural, biologically active components necessary for the body’s self-regulation. In developed regions such as Japan, the United States, and the European Union, dietary supplements are widely used to correct nutritional deficits and prevent disorders and diseases caused by geographic, environmental, emotional, and other factors [9,10].

Interest in dietary supplements continues to grow, yet many issues remain unresolved: methodological approaches for scientific research, technological challenges constraining production schemes, and procedures for substantiating complex (rather than single-component) supplements remain underdeveloped. Modern analytical support ensuring production safety and preventing adulteration is lacking. Delivery forms also require improvement, as criteria such as ease of consumption and swallowing are not always considered [11].

Research attention is increasingly directed toward raw materials for dietary supplements, contributing to the development of new industries and import substitution of both raw materials and final products. This will support the establishment of processing facilities and technological innovation in the agro-industrial sector. The use of modern methods for producing dietary supplements creates real opportunities for the rational and efficient use of raw materials and the development of immunomodulatory products.

Thus, developing dietary supplements using cereals, vegetables, and plant raw materials with immunomodulatory properties is not only scientifically relevant but also an important state task. Hence, this study targets increasing the production of immunomodulatory dietary supplements by improving the selection of main and auxiliary raw materials for supporting immune function.

This study targets immunomodulatory compositions due to the growing impact of natural disasters and emotional stress: the creation of technologically advanced complex dietary supplements capable of strengthening the immune system is an urgent issue.

The scientific novelty lies in developing a dietary supplement technology using modern processing methods, incorporating cereals (sprouted oats and barley), Jerusalem artichoke, and plant raw materials (licorice root) with immunomodulatory properties.

The tubers and aboveground parts of Jerusalem artichoke (*Helianthus tuberosus* L.) possess both nutritional and therapeutic characteristics. Jerusalem artichoke is used as a functional food, a source of biologically active compounds, and a raw material for producing biofuels. It is rich in inulin, an indigestible polysaccharide that serves as a prebiotic for gastrointestinal health. Historically, it has been used worldwide as a dietary supplement, to relieve pain, reduce swelling, strengthen immunity, and treat skin wounds in folk medicine. It contains numerous bioactive compounds such as phenolic acids, coumarins, and flavonoids with antioxidant, antimicrobial, and anti-inflammatory properties. However, the literature on its antidiabetic, anticancer, fungistatic, antiviral, and anti-obesity properties remains limited [12].

Grain sprouting is a sustainable and environmentally friendly method for increasing naturally occurring bioactive compounds such as GABA and polyphenols. These compounds can be used in functional foods and nutraceuticals aimed at improving insulin sensitivity and preventing metabolic disorders. Their natural origin and health benefits align with ongoing efforts to develop safe, food-based disease-prevention strategies. During grain germination, endogenous enzymatic systems activate, resulting in significant changes in both macronutrient and micronutrient profiles [13].

Liquorice, the root and rhizome of several species of the genus *Glycyrrhiza* L. (Leguminosae), is widely used in medicine, food production, animal feed, and cosmeceuticals. Liquorice exhibits numerous pharmacological effects, including activity similar to adrenal cortex hormones, as well as beneficial influences on the digestive, immune, cardiovascular, and nervous systems. In the food industry, it is used as a natural sweetener and flavoring agent in various products, particularly beverages [14].

Its broad applicability is largely attributed to the presence of triterpenoid saponins, such as glycyrrhizin, and flavonoids, many of which exist as prenylated or glycosylated derivatives. Glycyrrhizin, which constitutes about 5–10% of liquorice root, is a particularly potent sweetener—approximately 50 times sweeter than sucrose. Although the flavonoid content of liquorice is generally below 1% and varies by species, origin, growing conditions, and processing methods, these compounds possess substantial potential for the development of functional foods and health-promoting products [15,16].

The biological activity of these constituents underscores liquorice’s value as a nutraceutical ingredient, providing not only sweetness but also additional health benefits. Continued research on liquorice and its bioactive components is expected to further reveal its potential and contribute to improved protection of human and animal health [17,18,19]. In recent years, the extensive utilization of liquorice resources has attracted significant attention due to their applications in disease treatment, the production of health-oriented food, and various industrial sectors [20].

In summary, Jerusalem artichoke, sprouted oats, sprouted barley, and liquorice root exhibit prebiotic, immunomodulatory, and hypoglycaemic properties. The purpose of this study is to develop a complex biologically active supplement based on these ingredients, to substantiate the selection of raw materials, optimize their proportions, and evaluate the technological characteristics of the resulting composition.

## 2. Materials and Methods

We developed the technology based on standardized physiological requirements for various population groups for nutrients and energy, as well as on the concept of balanced and functional nutrition. The objects of the study were sprouted spring oats, sprouted spring barley, Jerusalem artichoke, and liquorice root.

All raw materials were obtained from certified suppliers in Kazakhstan. Spring oats (*Avena sativa* L., variety “Aman”) and spring barley (*Hordeum vulgare* L., variety “Arna”) were purchased from a local agricultural seed center and met the requirements of GOST 28672-2018 [21] for grain intended for food processing. Jerusalem artichoke tubers (variety “Skorospelka”) and dried liquorice root (*Glycyrrhiza glabra* L., pharmacopoeial grade) were sourced from accredited producers and complied with national food safety regulations.

Grain germination was conducted in a laboratory germination chamber (model: “SproutTech-120”) equipped with automated control of microclimate parameters. Before germination, oat and barley grains were washed, sorted manually to remove damaged kernels, and surface-sterilized by immersion in a 0.02% potassium permanganate solution for 2 min, followed by rinsing with distilled water.

The germination conditions were as follows:

- Temperature: maintained at 16–18 °C using a thermostatic control module;
- Humidity: 85 ± 2%, ensured through automatic misting nozzles;
- Light conditions: germination performed in the dark to ensure uniform sprouting;
- Duration: 36–48 h, with monitoring every 6 h;
- Air circulation: controlled ventilation system ensuring 0.3 m/s airflow.

Sprouting was continued until the sprouts reached a length of 2–10 mm. All conditions were continuously monitored using integrated sensors and recorded automatically. A control group consisting of non-germinated oat and barley grains was included to assess the effect of germination.

After germination, grains were dried at 40 °C in a convection drying oven (Memmert UF55, Memmert GmbH + Co. KG, Schwabach, Germany) until the moisture content fell below 10%. Jerusalem artichoke tubers were washed, peeled, sliced, and dehydrated at 45 °C. Liquorice root was milled directly in its dried form. All plant materials were milled using a laboratory mill (Cyclotec 1093) to achieve a homogeneous powder suitable for analysis and formulation.

All experiments were performed in triplicate (*n* = 3), and results are presented as mean ± SD. Statistical analysis was conducted using one-way ANOVA followed by Tukey’s post hoc test, with significance set at *p* < 0.05. Full ANOVA tables have been added to the revised manuscript.

The following analytical procedures and instruments were used:

- determination of germination energy and germination capacity according to GOST 10968-88 [22];
- moisture content of flour determined according to GOST 13586.5-2015 [23] using a Memmert drying oven;
- flour acidity determined according to GOST 27493-87 [24];
- quality analysis and organoleptic assessment of grain raw materials performed according to GOST 27558-87 [25];
- microbiological parameters determined according to GOST ISO 7218-2015 [26] using a LaminarFlow-BAV microbiological workstation;
- moisture determination according to GOST 24027.2-80 [27];
- fat content measured according to GOST 23042-86 [28] using a Soxhlet extraction unit;
- nitrogen and protein quantified by the Kjeldahl method according to GOST 25011-81 [29] and GOST R 50453-92 (ISO 937-78) [30] using a Kjeltec Auto 2300 analyzer (FOSS Analytical, Hillerød, Denmark);
- ash content determined according to GOST R 53642-2009 [31] using a muffle furnace Nabertherm L9/11 (Nabertherm GmbH, Lilienthal, Germany);
- magnesium and calcium content measured according to GOST EN 15505-2013 [32];
- phosphorus content determined spectrophotometrically according to GOST R 51482-99 (ISO 13730-96) [33];
- copper, iron, lead, and cadmium quantified by atomic absorption spectroscopy according to GOST 26931-86, GOST 26928-86, GOST 26932-86, and GOST 26933-86 [34,35,36,37] using an AAnalyst 200 atomic absorption spectrometer (PerkinElmer Inc., Waltham, MA, USA).

To determine the optimal composition of the biologically active supplement, four prototype formulations with different ratios of Jerusalem artichoke, sprouted oats, sprouted barley, and liquorice root were prepared. Ingredient proportions were optimized based on nutritional composition, functional parameters, and organoleptic characteristics. ANOVA confirmed no significant differences in inulin content among formulations (*p* = 0.61), allowing flexibility in raw material ratios for technological adaptation.

The obtained results are consistent with previously published data on the biochemical enhancement of sprouted grains, including increased enzymatic activity, improved amino acid profile, elevated antioxidant capacity, and reduction in anti-nutrients. Additional comparative analysis with recent research on plant-based immunomodulatory supplements has been included in the revised manuscript to highlight the novelty and relevance of the developed formulation.

### 2.1. Determination of Powder Flowability by Slope Angle Determination

A method widely used in the pharmaceutical and food industries is to measure the slope angle of a cone formed by powder poured onto a flat, level surface: the smaller (i.e., shallower) the slope angle, the higher the material’s flowability.

According to the classification adopted in the US Pharmacopoeia USP <1174> “Powder Flow” and the European Pharmacopoeia (EP, section 2.9.36), the normative values of the angle of natural slope are interpreted as follows (Table 1).

### 2.2. Determination of Bulk and Compacted Powder Density Using the Hsiang Tai PT-20 Tester

**Figure 1:**
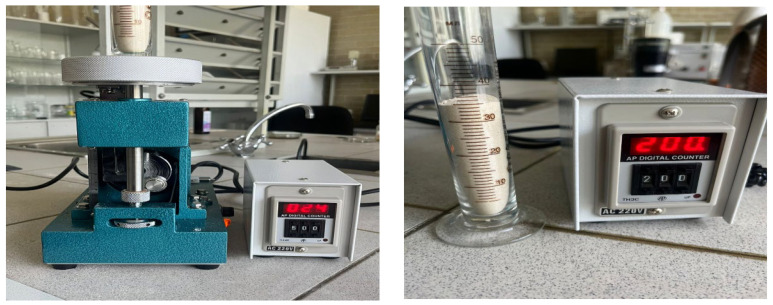
Hsiang Tai PT-20 Tester.

For each powder sample (control and experimental with the addition of a complex food additive), tests were performed on a Hsiang Tai PT-20 device (Hsiang Tai Machinery Industry Co., Ltd., Taichung, Taiwan) (Figure 1), following the manufacturer’s instructions. A sample weighing 15.00 g was placed in the device’s measuring cylinder, and its initial (bulk) volume was recorded. Next, the sample was compacted by 500 taps; at the end of the cycle, the compacted volume was recorded.

Loose density and tapped density are calculated using the formulas:

$$\rho_{v}=\frac{m}{V_{V}},\;\rho_{T}=\frac{\rho }{V_{T}}$$

where *m* is the weight of the suspension (recorded in grams), and *V_V_* and *V_T_* are the volumes before and after compaction, recorded in cm^3^ (mL).

### 2.3. Powder Encapsulation

**Figure 2:**
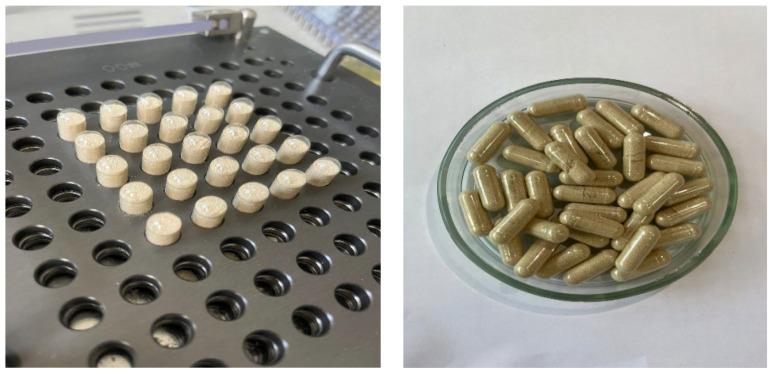
Powder encapsulation on a semi-automatic capsule-filling machine.

For the selected dietary supplement sample, encapsulation was performed using a Semi-Auto Capsule Filling Machine (matrix capacity 100 capsules) (Figure 2). Gelatine capsules were placed in the lower part of the matrix, after which a weight of powder was manually added, and the surface was leveled with a scraper. Next, the upper part of the matrix was combined with the lower part, and the capsules were pressed together until they were completely closed.

### 2.4. Determination of the Water Holding Capacity of Four Samples

The method is based on the GOST 7636-85 [38] method for centrifugation and is adapted for vegetable powders.

**Figure 3:**
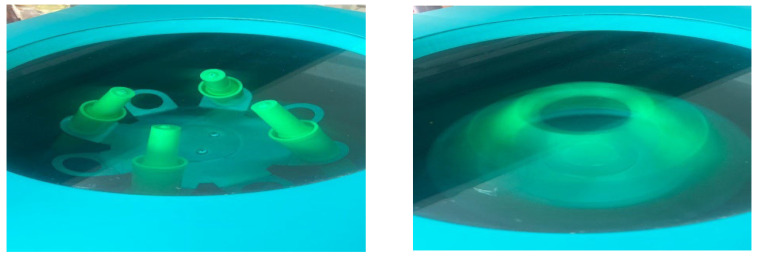
Determining water holding capacity by centrifugation.

1. A sample of powder (1 g) was placed in a centrifuge tube.
2. 10 mL of distilled water was added, and the mixture was left at room temperature for 30 min.
3. The tubes were centrifuged for 15 min (Figure 3).
4. The separated water was drained, and the remaining sediment was weighed.
5. The water holding capacity (WHC) was calculated using the formula:

$$WHC=\frac{m_{2}-m_{1}}{m_{1}}$$

where *m*_1_ is the mass of the dry powder, and *m*_2_ is the mass of the wet precipitate after centrifugation and draining.

The samples were characterized by moisture retention, flowability, and hygroscopicity. Using Statistica 14.2.0.18, the relationship between the proportion of components and the inulin content was determined by Pearson’s correlation analysis. A one-factor analysis of variance (ANOVA) was also performed to assess the effect of changes in Jerusalem artichoke dosage on the inulin level in the composition. The threshold for statistical significance was set at *p* < 0.05.

## 3. Results

In recent decades, interest in functional nutrition and the use of plant-based biologically active additives (dietary supplements) for disease prevention and health maintenance has steadily increased [39]. Plant-derived components with high biological activity and low toxicity are widely used in the development of functional food products.

Sprouted cereals and medicinal plants are particularly promising sources of bioactive compounds, as germination significantly increases the content of enzymes, vitamins, antioxidants, and other beneficial substances. The germination process promotes enzyme activation, the breakdown of complex carbohydrates, and a reduction in antinutritional factors, ultimately improving the digestibility and bioavailability of micronutrients [40].

One of the priorities in the development of functional supplements is supporting metabolic health, including the prevention and adjunctive management of diabetes mellitus. According to the World Health Organization, the number of individuals with diabetes continues to rise, highlighting the urgent need for natural remedies capable of improving carbohydrate metabolism [41,42]. One of the key factors contributing to diabetes development is an unbalanced diet—specifically, excessive consumption of refined carbohydrates and insufficient intake of dietary fiber. Therefore, functional components that help normalize metabolic processes are of particular importance. Inulin, a soluble dietary fiber, is characterized by a low glycaemic index and the ability to lower blood glucose and cholesterol levels by modulating lipid metabolism and exerting antioxidant effects [43,44,45,46,47].

In the development of a dietary supplement intended for individuals with diabetes and for immunomodulatory purposes, four components were selected: sprouted oats, sprouted barley, liquorice root, and Jerusalem artichoke. The selection of these ingredients is based on their scientifically proven properties aimed at supporting carbohydrate metabolism, reducing blood sugar levels, and strengthening the body’s immune functions. Each component performs specific physiological roles, while their combined use provides a more pronounced synergistic and therapeutic effect.

The inclusion of inulin is justified by its ability to support metabolic regulation and enhance the body’s immune defense mechanisms. To meet the daily requirement for inulin, a person must consume 400–500 g of vegetables and fruits, which is not always feasible. Jerusalem artichoke, one of the richest natural sources of inulin, contains highly bioavailable polysaccharides; various technological approaches are used to extract inulin while preserving its biological properties.

When selecting raw materials, not only inulin content but also bioavailability, pharmacological properties, and component compatibility must be considered.

Sprouted cereals such as oats and barley possess high nutritional and biological value due to increased concentrations of enzymes, vitamins, and other bioactive molecules. Germination—the process through which a dormant seed transitions to an active growth phase—is one of the most effective methods for enhancing the nutritional quality of grain raw materials [48,49]. Germination improves mineral bioavailability, reduces antinutritional factors, and enhances the amino acid profile. Biochemical transformations occurring during sprouting lead to the formation of simple sugars and structural modification of starch, which can positively influence metabolic processes in individuals with diabetes.

Liquorice root has long been used in traditional and modern medicine due to its antioxidant, anti-inflammatory, and immunomodulatory effects. Its active components—glycyrrhizic acid and various flavonoids—help reduce inflammation, stabilize blood sugar levels, and improve adrenal gland function. The use of liquorice in dietary supplements for individuals with diabetes is supported by its ability to regulate carbohydrate metabolism and enhance the body’s adaptive responses [48].

Thus, the presented data suggest that oats, barley, liquorice root, and Jerusalem artichoke are highly promising crops for dietary and therapeutic applications. Their unique biochemical composition allows them to beneficially modulate metabolic processes and prevent or alleviate metabolic disorders.

Sprouted oats and sprouted barley were selected as the primary research objects. Under laboratory conditions, the grains were germinated using a controlled germination system, where they were evenly moistened with warm water. The dependence of germination time on temperature was established: optimal germination occurred at 16–18 °C over a period of 36–48 h.

A germination analysis of sprouted oats and barley was carried out (Table 2).

**Figure 4:**
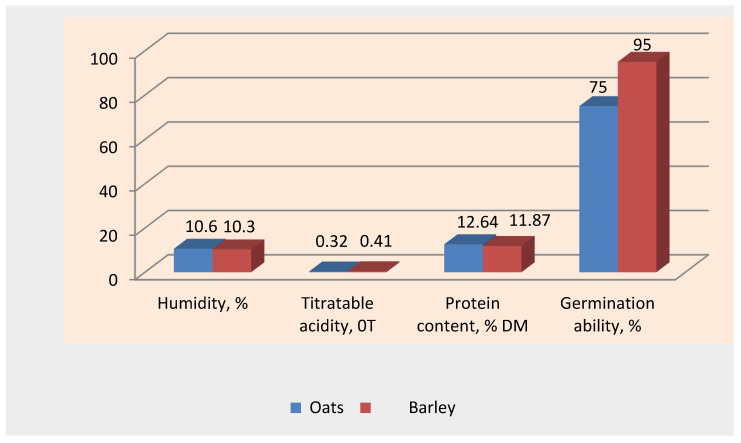
Characteristics of sprouted grains.

From Table 2, it can be seen that increasing the germination time resulted in markedly longer shoots, a greater germination rate, which reached 95% after 48 h, and increased moisture. Germination is faster in barley than in oats. To prevent spoilage, the sprouted grain was dried in the “Home Station 2” chamber at a temperature of 43–45 °C for 12–14 h to a humidity of 10–12%. The main characteristics of sprouted oat and barley grains are shown in Figure 4.

The moisture contents were similar: 10.6% for oats and 10.3% for barley, which indicates optimal germination conditions for both types of grain. These data show that the drying process after germination was successful and ensured the stability of their structure. Oats have a higher protein content, and barley has a higher germination capacity.

The titrated acidity of sprouted oat grains (0.32 °T) is lower than that of barley (0.41 °T), which may indicate a milder nature of oats in terms of acidity, which, in turn, may affect their susceptibility to various storage and processing conditions.

The protein content in sprouted oat grains (12.64%) was higher than in barley (11.87%). This confirms that oats are a more protein-rich grain, which is important for their use in food additives, especially for people with high protein requirements.

Barley’s higher germination capacity (95% vs. 75% for oats) is important for the efficient use of grain in the process of obtaining germinated products.

The physico-chemical parameters, vitamin content, macro- and microelement content, and amino acid composition were determined for the sprouted grains, and also for crushed Jerusalem artichoke and liquorice root (Table 3).

Sprouted oats (12.64%) and barley (11.87%) contain significant amounts of protein compared to the Jerusalem artichoke (8.32%). This indicates the suitability of these grains as protein sources, which can be useful for maintaining a normal metabolism, especially for people with diabetes. Jerusalem artichoke, in turn, is distinguished by its high fiber content (24.65%), which improves the functioning of the digestive tract and can be useful for detoxification of the body. The vitamins contained in these materials are also diverse: Jerusalem artichoke is rich in vitamin C (31.95 mg/100 g), while oats and barley contain B vitamins such as B1, B3, and B6, which make these foods valuable for maintaining overall health. It is also worth noting that oats and barley contain minerals such as magnesium, potassium, and calcium, which are important for the normal functioning of the cardiovascular system and metabolism. This highlights their potential for inclusion in diets aimed at improving metabolism and maintaining overall health, especially for people who are deficient in these trace elements.

The measurements emphasize the importance of the studied products as valuable sources of nutrients that can be used to develop new dietary supplements. The germination process increases the bioavailability of vitamins and minerals, improving their absorption, and also increases antioxidant activity, which helps protect cells from oxidative stress [50].

The daily fiber requirement for diabetics is 50 g. Sprouted oats, barley, liquorice root, and Jerusalem artichoke contain, respectively, 10.03, 8.62, 3.47, and 24.65 g fiber/100 g, which cover 20.06%, 17.24%, 6.94%, and 49.3%, respectively. Thus, sprouted oats, barley, and liquorice root are good sources of fiber and amino acids (Table 4).

Table 4 demonstrates that the sprouted grains contain significant amounts of essential amino acids such as lysine, valine, threonine, and phenylalanine, which play key roles in maintaining the immune system, tissue regeneration, and other vital processes. In particular, the levels of lysine and phenylalanine are significantly higher than in unsprouted oats and barley. These measurements are consistent with other studies confirming that germination increases the essential amino acid content in grain. It is also worth noting that the high levels of arginine and methionine observed confirm these grains’ potential to support the body’s metabolic and immune functions. The results obtained can be useful not only for people who monitor their diet, but also for athletes, as well as those who need an additional source of amino acids to restore and improve the overall condition of the body.

Jerusalem artichoke, in turn, is rich in inulin and minerals, which make it useful for maintaining the digestive system and metabolism [51].

Microbiological parameters of raw materials (Jerusalem artichoke, sprouted oats, sprouted barley, liquorice root, oats, barley) were determined (Table 5).

The microbiological data in Table 5 demonstrate that all the studied samples (Jerusalem artichoke, oats, barley, and liquorice root) comply with sanitary standards, since the microbiological indicator counts (TMAFAM, EC group bacteria, yeast, and molds) fall within acceptable values. This confirms that the raw materials have been properly processed and comply with safety standards for food and dietary supplements.

The results show that sprouted oats and barley are characterized by a high content of vitamins and essential amino acids, which makes them valuable for nutrition, especially in conditions of a limited diet. These grains are an excellent source of essential substances for maintaining overall health and can be recommended as a supplement to diets. Jerusalem artichoke, in turn, is rich in inulin and minerals, which help improve the functioning of the digestive system and metabolism. It also has a positive effect on regulating blood sugar levels, which makes it especially useful for people with diabetes.

In addition, the germinated seeds of each crop have a unique composition, including amino acids, polysaccharides, and trace elements, which allows them to have a targeted health-improving effect. They can be recommended for people suffering from various diseases, including metabolic disorders.

Grain germination helps to increase the bioavailability of nutrients, which helps to improve the absorption of vitamins and minerals. Sprouted grains, Jerusalem artichoke, and liquorice root have antioxidant properties, protecting cells from oxidative stress. Under conditions of stress, malnutrition, and adverse environmental factors, the importance of using dietary supplements with herbal components increases, as they can effectively maintain health and prevent diseases such as diabetes and metabolic disorders.

The measurements obtained show that the germination process significantly increases the bioavailability of nutrients, contributing to improved absorption of vitamins and minerals by the body. Sprouted grains, Jerusalem artichoke, and liquorice root have antioxidant properties, which have an important effect on protecting cells from oxidative stress.

When developing a dietary supplement from these ingredients, not only was the composition taken into account, but also the interaction of the components, since their combination enhances or weakens the beneficial properties of each other (Table 6).

Table 6 shows how the proposed dietary supplement formulation accounts for the compatibility of its components and their mutual enhancement of useful properties. The chosen combination allows for metabolic support, normalization of intestinal microflora, and reduction in glycaemic load, which is especially important for people with diabetes mellitus.

Four recipes for composing a biologically active additive (dietary supplement) from these four ingredients were studied (Table 7). To assess the functional value and dosage of each composition, the inulin content was calculated from its proportion in each component: 15%, 10%, and 5% Jerusalem artichoke, sprouted oats, and sprouted barley, respectively; liquorice root lacks inulin [52].

An organoleptic evaluation of various dietary supplement compositions was performed (Table 8). Data on the appearance, color, odor, taste, texture, and moisture content of the samples are presented. Based on the obtained results, the sample with the optimal composition of the dietary supplement according to organoleptic characteristics was identified.

According to Table 8, Recipes 1 and 3 demonstrate the least favorable organoleptic characteristics. Recipe 1 has a rough texture, a strong vegetative odor, an unpleasant aftertaste, and elevated moisture content (7.2%), which may negatively affect its storage stability. Recipe 3 exhibits a pungent odor, a bitter–sour taste, and an uneven consistency, making it less preferable.

Recipe 2 shows intermediate characteristics, with a tart taste, medium friability, and moderately reduced moisture (6.9%). Recipe 4 exhibits the most favorable organoleptic profile: homogeneous texture, pleasant herbal aroma, moderately sweet taste, and the lowest moisture content (6.5%), which contributes to its stability during storage.

The best organoleptic performance was demonstrated by Recipe 4, which contained 60 g Jerusalem artichoke, 12.5 g sprouted oats, 12.5 g sprouted barley, and 15 g liquorice root.

To justify the final formulation of the dietary supplement, several component ratios were evaluated. The main selection criteria included:

− physical suitability of the mixture (flowability, moisture retention capacity);
− functional balance ensuring sufficient intake of prebiotic substances, dietary fiber, and biologically active compounds.

The mechanical properties of the powder are essential for ensuring uniform distribution of active ingredients, accurate dosing, and stable product quality under industrial conditions. Finely ground powder enhances bioavailability and facilitates faster release of active components during digestion. Additionally, physical properties directly influence storage behavior, including resistance to caking, prevention of nutrient degradation, and preservation of organoleptic characteristics. Therefore, these measurements form the basis for designing dietary supplements with stable performance, safety, and consumer acceptability [53].

Water retention capacity (WHC) plays an important role in evaluating the behavior of supplements upon moisture interaction and their swelling, fluidity, and homogeneity. Although no official GOST standards regulating WHC for food powders exist, scientific and industrial practice widely relies on this indicator for characterizing plant-based raw materials and functional additives. According to the literature, typical WHC ranges from 0.5 to 1.0 g water per gram of dry matter for most vegetable powders and botanical additives [39,40]. Higher WHC (>1.0 g/g) is characteristic of fibrous or gel-forming materials (pectin, guar gum), while lower values (<0.5 g/g) occur in starch- and protein-rich powders.

Bulk and compacted density are also important indicators of flowability, caking tendency, and behavior during dosing and storage. Comparing loose bulk density to compacted density after simulated mechanical stress makes it possible to assess the ease of processing and long-term stability of consumer properties.

To address the reviewer’s comment, additional stability testing was performed. The optimized formulation (Recipe 4) underwent a three-month accelerated storage study at controlled conditions (25 °C, 60% RH). Organoleptic properties—appearance, smell, taste, color, and texture—as well as moisture content, were monitored at 0, 30, 60, and 90 days.

Throughout the storage period, no significant changes in odor, taste, or color were detected, and the product retained a loose, non-caking consistency. Moisture increased only slightly (from 6.5% to 6.7%), remaining within acceptable limits. These results confirm that Recipe 4 maintains stable organoleptic properties over time and is suitable for practical application and long-term shelf-life.

The flowability was assessed via the natural cone slope angle (Table 9) when the powder is freely poured onto a horizontal surface. The slope angle reflects the friction and adhesion between particles and, therefore, indirectly characterizes the flowability of the material [41,42].

According to Table 9, only Recipe 4 falls into the preferred water retention capacity range for biologically active additives. The moderate moisture absorption will promote swelling in the gastrointestinal tract, improving digestion and creating a feeling of fullness. This level of water retention does not cause excessive gelation and preserves the convenience of administration and uniform release of active ingredients, which is especially important for powder and capsule delivery.

All four recipes fall within the standard ranges for bulk (0.5–1.1 g/mL) and compacted (0.6–1.3 g/mL) densities. However, the differences between them demonstrate the ingredients’ differing influences on the composite powder properties. Recipe 1 is characterized by the lowest densities (0.50 and 0.62 g/mL), which indicates a looser powder structure and lower compaction capacity. Recipe 3 also shows satisfactory values (0.53 and 0.67 g/mL), but its values remain at the lower limit of the norm. This may be attributed to the increased content of sprouted oats and barley, which have a lower density.

The best densities are achieved by Recipes 2 and 4: 0.58 and 0.72 g/mL and 0.60 and 0.74 g/mL, respectively, which are important for the stability of dosing and further use of dietary supplements.

Recipes 2 and 4 also demonstrated the best flowability, with cone slopes of 35° and 34°, respectively, indicating ease of dosing and packaging.

Considering all of the measurements, the best option is Recipe 4 (a 60:12.2:12.5:15 mass ratio of Jerusalem artichoke, sprouted oats, sprouted barley, and liquorice root). This composition is characterized by a high water retention capacity, which indicates a significant content of dietary fiber and the ability to swell, as well as good flowability for manufacturability. Recipe 4 also best demonstrated the best organoleptic properties.

To assess the contribution of individual plant components to the formation of inulin content in the composition of the developed dietary supplement, a correlation analysis was performed. The amounts of Jerusalem artichoke, sprouted oats, sprouted barley, and liquorice root (g) were considered as independent variables, and the inulin content (%) was considered as dependent. The results of the analysis are shown in Table 10.

**Figure 5:**
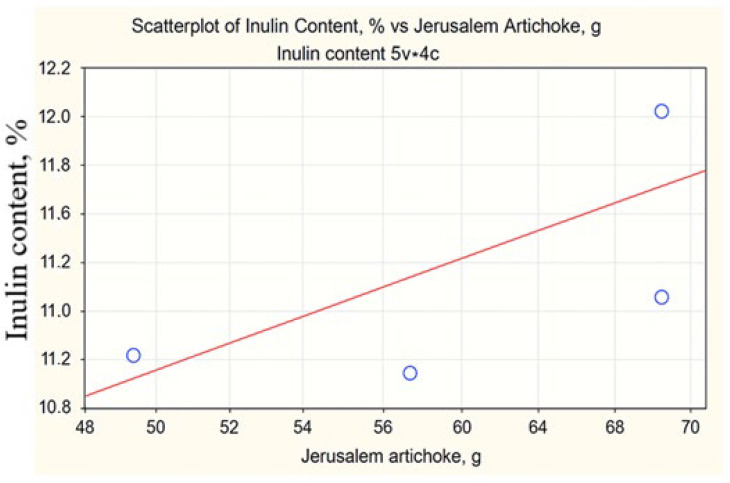
Dependence of inulin content on the Jerusalem artichoke mass in the four recipes.

**Figure 6:**
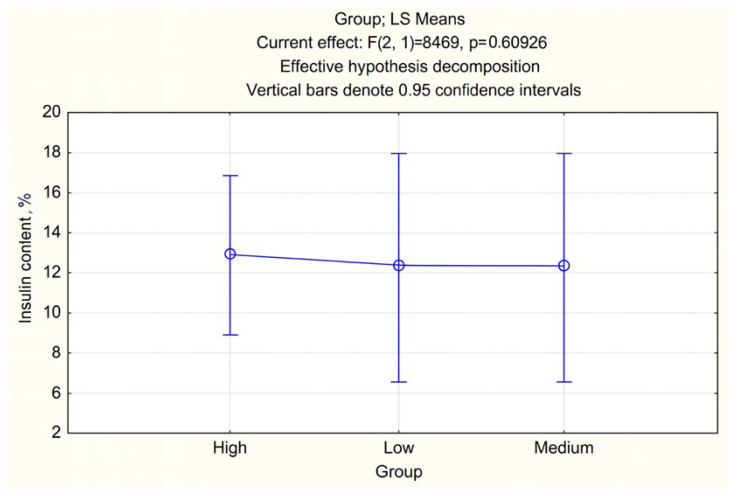
The average inulin content (%) in samples with different levels of Jerusalem artichoke addition (data presented as LS Means ± 95% CI; ANOVA, *p* > 0.05).

The correlation analysis showed that Jerusalem artichoke mass (*r* = 0.670) has the greatest positive relationship with inulin content, while the masses of oat (*r* = −0.396), barley (*r* = −0.389), and liquorice root (*r* = −0.095) did not demonstrate substantial correlations with this indicator. A statistically significant relationship was found only between oats and barley (*r* = 0.989, *p* < 0.05), which is explained by their parallel increase in the formulations (Figure 5 and Figure 6).

When comparing formulations with different amounts of Jerusalem artichoke (50, 60, and 70 g), no statistically significant differences in inulin content were observed (*p* = 0.61). This indicates that reducing the Jerusalem artichoke proportion does not significantly affect the inulin level, allowing the formulation to be optimized from both economic and technological perspectives.

Taking into account the results of the correlation and variance analyses, as well as the earlier physicochemical and organoleptic evaluations, Recipe 4 was identified as the optimal formulation. Given these findings and the technological advantages of Sample 4, an analysis of variance (ANOVA) was additionally performed to determine whether the Jerusalem artichoke dosage could be further reduced without a meaningful loss of functional properties.

The qualitative and microbiological characteristics of the dietary supplement samples were subsequently assessed at the Research Laboratory for the Quality and Safety Assessment of Food Products of Almaty Technological University JSC (Table 11 and Table 12).

According to Table 11, Recipe 1 contains the largest amount of dietary fiber, which indicates its high value for normalizing the gastrointestinal microflora. Recipe 2 exhibits higher mineral content and higher acidity, which may offer a positive effect on digestibility, but its protein and vitamin levels were lower than those of the other formulations. Recipe 3 had minimal values for most of the parameters studied, indicating an insufficiently balanced formulation and poor nutritional value.

The most promising is Recipe 4, which offers the greatest protein content, high levels of vitamin E, and sufficient amounts of minerals. This composition provides both nutritional and functional value, making it the most suitable for further use in enriching flour for baked goods.

The highest arginine content is observed in Recipe 1 (2.388 ± 0.955%), while the lowest is in Recipe 3 (1.249 ± 0.499%). Recipe 1 also had the highest lysine content (1.298 ± 0.441%), while the lowest level was found in Recipe 2 (0.331 ± 0.113%).

It should be noted that the highest proline values are typical for Recipes 2 and 4 (2.240 ± 0.582% and 2.460 ± 0.640%, respectively). The phenylalanine content was highest in Recipes 3 and 4 (0.832 ± 0.250% and 0.846 ± 0.254%).

Thus, each of the recipes has its own characteristic amino acid profile: Recipe 1 is characterized by an increased content of arginine and lysine, which indicates its high biological value, whereas Recipes 2 and 4 are distinguished by the level of proline, and Recipes 3 and 4 by substantial phenylalanine.

Microbiological parameters were determined for each recipe, as shown in Table 13, and were satisfactory and met the requirements of SanPiN.

Considering all the analyses undertaken, we conclude that all four recipes comply with the quality standards and offer high nutritional value and a functional orientation to address deficiencies in essential amino acids and macro- and microelements, as well as to improve metabolic processes. The integration of multiple selected ingredients into dietary supplements opens new prospects for creating functional products with immunomodulatory and antioxidant properties.

## 4. Discussion

The results obtained confirm the feasibility of developing an immunomodulatory, biologically active food supplement based on a synergistic combination of sprouted grains (oats, barley), Jerusalem artichoke, and liquorice root. Each component provides distinct nutritional and functional benefits, and their combined use enhances the overall physiological potential of the formulation.

A comparison with existing research shows that the high protein content and improved amino acid profile of sprouted oats (12.64%) and barley (11.87%) are consistent with previous studies demonstrating enhanced digestibility, increased essential amino acids, and reduced levels of anti-nutritional factors during germination [41,50]. Petrova et al. (2020) [41] also reported that germination increases antioxidant activity and improves mineral bioavailability, findings that align with ours and support the use of sprouted grains as effective metabolic modulators in plant-based supplements.

Jerusalem artichoke served as the primary source of dietary fiber (24.65%) and vitamin C (31.95 mg/100 g), comparable to values reported in recent studies on inulin-rich prebiotic plant materials [43,44,45,46,47]. The strong correlation between inulin content and the Jerusalem artichoke proportion (*r* = 0.67) is consistent with the literature, which describes inulin as a key compound involved in glycaemic control, lipid metabolism regulation, and gut microbiota modulation. Several works highlight the synergistic enhancement of inulin’s prebiotic action when combined with antioxidant-rich plant materials, further supporting its applicability in multifunctional formulations [48,49].

Liquorice root contributed glycyrrhizic acid and vitamin B2 (0.77 mg/100 g), consistent with literature describing its potent immunomodulatory, antiviral, anti-inflammatory, and adaptogenic functions. Numerous studies confirm that glycyrrhizic acid regulates pro-inflammatory cytokines (TNF-α, IL-6), supports antioxidant defense, and modulates the hypothalamic–pituitary–adrenal axis, thereby strengthening immune and metabolic resilience [50,51]. These findings substantiate the rationale for including liquorice as a synergistic immunomodulatory component.

Four formulations were prepared with varying proportions of ingredients. The optimization process was based on a combined assessment of nutritional value, organoleptic characteristics, technological properties, and statistical analysis. While a full factorial Design of Experiments (DoE) was not applied, the selection was data-driven: ANOVA testing demonstrated no statistically significant differences in inulin content across formulations (*p* = 0.61), indicating that the functional fiber contribution remains stable even with adjusted ingredient ratios. Recipe 4 (60 g Jerusalem artichoke, 12.5 g oats, 12.5 g barley, 15 g liquorice root) displayed optimal powder flowability (slope angle 34°), moisture retention (0.701 g/g), and sensory acceptability, suggesting superior processing and storage stability.

While literature supports the immunomodulatory properties of each component individually, the synergistic effect of their combination requires further biological verification. No in vitro or in vivo assays were conducted within this study; therefore, conclusions about combined immunomodulatory action are based on biochemical composition rather than physiological testing [52,53,54]. Future studies should include:

- in vitro assays (macrophage activation, cytokine profiling, antioxidant capacity);
- in vivo models of metabolic dysregulation or immune suppression;
- microbiome analysis to evaluate prebiotic synergy.

Such studies would substantiate the hypothesized synergistic mechanisms and confirm the supplement’s efficacy beyond compositional analysis.

Based on the biochemical characteristics of the ingredients and existing literature, the mechanism of action may involve several pathways:

1. Metabolic regulation:
  − inulin improves glucose tolerance and insulin sensitivity;
  − sprouted grains enhance amino acid availability, supporting muscle metabolism and glycaemic stability;
  − liquorice flavonoids modulate carbohydrate metabolism and reduce oxidative stress.
2. Immunomodulation:
  − glycyrrhizic acid regulates immune signaling pathways (NF-κB, IL-6, IL-1β);
  − antioxidant compounds in sprouted grains and Jerusalem artichoke reduce inflammatory stress;
  − prebiotic fibers enhance gut microbiome diversity, promoting mucosal immunity.
3. Synergy:
  − prebiotic fibers amplify the bioactivity of flavonoids;
  − germinated grain enzymes improve micronutrient bioavailability, enhancing the body’s adaptive responses.

Overall, the developed formulation is aligned with global trends toward plant-based, multifunctional immunomodulatory supplements. While the compositional data support its nutritional and functional potential, biological validation will be required to fully substantiate its immunomodulatory synergistic effects.

## 5. Conclusions

The study demonstrated that sprouted oats and sprouted barley possess a high content of vitamins and essential amino acids, making them valuable ingredients for enriching the diet, particularly under conditions of limited nutrition. Germination significantly enhanced the nutritional profile of both grains: protein levels increased to 12.64% in oats and 11.87% in barley, while essential amino acids such as lysine (1.42%) and arginine (1.57%) were elevated. These results confirm the effectiveness of germination as a method for improving the biological value of grain raw materials. Sprouted oats and barley may therefore be recommended as functional components that support general health.

Jerusalem artichoke was confirmed to be a rich source of dietary fiber and bioactive compounds. Its high fiber content (24.65%) provides nearly half of the recommended daily intake for individuals with diabetes, while its vitamin C content (31.95 mg/100 g) contributes to the antioxidant capacity of the supplement. These properties highlight the potential of Jerusalem artichoke to support glycaemic control and antioxidant protection.

Liquorice root contributed both immunomodulatory properties and improved organoleptic characteristics of the formulations. Its functional activity is associated with the presence of glycyrrhizic acid and vitamin B2 (0.77 mg/100 g), which enhance adaptive responses in the body and impart a more pleasant taste profile to the supplement.

The optimal composition of the dietary supplement—containing Jerusalem artichoke powder, sprouted oats, sprouted barley, and liquorice root—was identified. Comparison of four prototype formulations showed that Recipe 4 exhibited the most balanced characteristics. Its nutritional and technological indicators (11.97% protein, 9.24% fiber, 57.75 mg/100 g vitamin E, moisture-holding capacity 0.701 g/g, and a flowability slope angle of 34°) demonstrate a favorable combination of functional value and mechanical stability. These parameters support the feasibility of using this formulation to enhance both the quality and functional properties of food products.

Microbiological evaluation confirmed the safety of the samples (TMAFAM < 3 × 10^4^ CFU/g, yeast < 6 CFU/g, no mold detected), indicating compliance with established food safety standards.

Statistical analysis revealed that Jerusalem artichoke serves as the primary source of inulin in the formulations (*r* = 0.67). According to the regression model, an additional 10 g of Jerusalem artichoke increases the inulin content by 0.35%. However, ANOVA results (*p* = 0.61) showed no significant differences when varying the Jerusalem artichoke content from 50 to 70 g, suggesting flexibility in raw material proportions without compromising functional effectiveness. This finding allows for cost-efficient formulation optimization.

Overall, the developed dietary supplement represents a promising functional product aimed at supporting metabolic health and assisting in the prevention of diabetes mellitus. The integration of natural plant ingredients with technologically advanced processing ensures the preservation of biologically active compounds and enhances the potential health benefits of the final product.

## Figures and Tables

**Table 1:** Flowability index.

Slope Angle (°)	Flowability
25–30	excellent
31–35	good
36–40	satisfactory
41–45	mediocre
46–55	bad
>55	very bad

**Table 2:** Germination characteristics of oat and barley.

Duration of Germination	Humidity (%)		Acidity (°C)		Sprout Length (mm)		Germination Rate (%)	
	Oats	Barley	Oats	Barley	Oats	Barley	Oats	Barley
24 h	45.5	45.4	0.28	0.32	2	4	20	40
36 h	47.5	48.1	0.32	0.36	6	8	40	80
48 h	47.8	48.3	0.34	0.43	8	10	75	95

**Table 3:** Characteristics of raw materials.

	Jerusalem Artichoke	Oats	Sprouted Oats	Barley	Sprouted Barley	Liquorice Root
*Physico-chemical parameters*						
Mass fraction of protein, %	8.32 ± 0.09	11.95 ± 0.16	12.64 ± 0.15	10.83 ± 0.14	11.87 ± 0.14	10.48 ± 0.12
Mass fraction of moisture, %	9.63 ± 0.13	9.89 ± 0.17	6.59 ± 0.10	10.21 ± 0.17	8.58 ± 0.13	7.1 ± 0.23
Mass fraction of ash, %	5.17 ± 0.02	3.37 ± 0.05	3.35 ± 0.04	2.77 ± 0.03	1.93 ± 0.04	6.245 ± 0.005
Mass fraction of fiber, %	24.65 ± 0.29	11.31 ± 0.13	10.03 ± 0.12	12.09 ± 0.16	8.62 ± 0.10	3.47 ± 0.04
Titrated acidity, °T	0.86 ± 0.019	0.422 ± 0.005	0.364 ± 0.008	0.508 ± 0.006	0.463 ± 0.011	1.521 ± 0.034
Foreign impurities	Not detected					
*Vitamin content of raw materials, mg/100 g*						
Vitamin E	0.912 ± 0.01	1.554 ± 0.02	2.61 ± 0.03	1.398 ± 0.019	1.01 ± 0.01	0.08 ± 0.001
Vitamin B_1_	0.365 ± 0.073	0.38 ± 0.075	0.472 ± 0.094	0.361 ± 0.07	0.294 ± 0.058	0.413 ± 0.082
Vitamin B_2_	0.274 ± 0.115	-	0.093 ± 0.039	-	0.114 ± 0.047	0.77 ± 0.032
Vitamin B_3_	8.21 ± 1.64	4.28 ± 0.86	1.38 ± 0.27	5.24 ± 1.05	2.69 ± 0.54	2.15 ± 0.43
Vitamin B_6_	-	2.46 ± 0.049	0.26 ± 0.05	0.317 ± 0.063	0.276 ± 0.055	0.103 ± 0.02
Vitamin C	31.95 ± 5.7	-	-	-	-	2.57 ± 0.46
*Mineral content, mg/100 g*						
Iron	1.83 ± 0.021	5.61 ± 0.07	10.27 ± 0.12	5.95 ± 0.08	4.64 ± 0.05	2.75 ± 0.03
Magnesium	63.89 ± 0.76	110.32 ± 1.54	126.10 ± 1.51	142.01 ± 1.98	89.61 ± 1.07	27.46 ± 0.33
Calcium	98.77 ± 1.18	120.07 ± 1.68	109.29 ± 1.31	105.16 ± 1.47	55.48 ± 0.66	80.25 ± 0.96
Potassium	1007.44 ± 12.09	463.24 ± 6.48	393.25 ± 4.72	459.72 ± 7.43	260.97 ± 3.13	315.02 ± 3.78
Phosphorus	424.46 ± 5.09	248.16 ± 3.47	337.21 ± 4.04	292.30 ± 4.09	210.79 ± 2.59	56.17 ± 0.67
Iodine	-	0.0033 ± 0.00001	0.008 ± 0.0001	0.004 ± 0.0001	0.0059 ± 0.0001	0.013 ± 0.0001

**Table 4:** Amino acid composition of raw materials.

	Jerusalem Artichoke	Oats	Sprouted Oats	Barley	Sprouted Barley	Liquorice Root
Arginine	0.964 ± 0.385	1.570 ± 0.628	1.267 ± 0.507	1.363 ± 0.545	1.097 ± 0.439	1.138 ± 0.455
Lysine	0.450 ± 0.153	1.221 ± 0.415	1.429 ± 0.486	1.107 ± 0.376	1.426 ± 0.485	1.138 ± 0.387
Tyrosine	0.418 ± 0.125	0.406 ± 0.122	0.747 ± 0.224	0.426 ± 0.128	0.695 ± 0.208	0.534 ± 0.160
Phenylalanine	0.610 ± 0.183	0.872 ± 0.262	0.747 ± 0.224	0.809 ± 0.243	0.621 ± 0.186	0.569 ± 0.171
Histidine	0.308 ± 0.15	0.523 ± 0.262	0.845 ± 0.42	0.468 ± 0.234	0.512 ± 0.256	0.391 ± 0.196
Leucine + isoleucine	0.675 ± 0.175	1.090 ± 0.283	1.104 ± 0.287	1.022 ± 0.266	1.060 ± 0.276	0.889 ± 0.231
Methionine	0.145 ± 0.049	0.148 ± 0.05	0.552 ± 0.188	0.183 ± 0.062	0.548 ± 0.186	0.462 ± 0.157
Valine	0.707 ± 0.283	0.959 ± 0.384	0.975 ± 0.390	0.894 ± 0.358	0.951 ± 0.380	0.889 ± 0.356
Proline	1.028 ± 0.267	0.828 ± 0.215	0.747 ± 0.194	0.766 ± 0.199	0.731 ± 0.190	0.747 ± 0.194
Threonine	0.482 ± 0.193	0.698 ± 0.279	0.845 ± 0.338	0.639 ± 0.255	0.877 ± 0.351	0.747 ± 0.299
Serine	0.803 ± 0.209	0.785 ± 0.204	0.715 ± 0.186	0.724 ± 0.188	0.695 ± 0.181	0.605 ± 0.157
Alanine	0.578 ± 0.150	0.741 ± 0.193	1.234 ± 0.321	0.681 ± 0.177	1.133 ± 0.295	1.032 ± 0.268
Glycine	0.675 ± 0.229	0.741 ± 0.252	0.780 ± 0.265	0.681 ± 0.232	0.768 ± 0.261	0.783 ± 0.266

**Table 5:** Microbiological parameters of raw materials.

	Jerusalem Artichoke	Sprouted Oats	Oats	Sprouted Barley	Barley	Liquorice Root
TMAFAM (CFU/g)	3 × 10^4^	3 × 10^4^	4 × 10^3^	7 × 10^3^	7 × 10^3^	4 × 10^4^
EC group bacteria (coliforms) in 1.0 cm^3^ of product	not detected					
Yeast (CFU/g)	5	2	6	6	5	1
Mold growth rate (CFU/g)	not detected					

TMAFAM—Total Mesophilic Aerobic and Facultative Anaerobic Microorganisms; EC Group Bacteria—Bacteria of the *Escherichia coli* group.

**Table 6:** Interaction of dietary supplement components.

Ingredient	Contribution to Dietary Supplement	Interaction with Other Components	Possible Risks
Jerusalem artichoke	A natural source of inulin improves intestinal microflora and promotes mineral absorption.	Enhances the bioavailability of minerals from sprouted cereals.	Large doses can cause bloating.
Sprouted oats	A source of dietary fiber, enzymes, and B vitamins.	Improves inulin absorption and lowers the glycaemic index.	Possible loss of enzymes during prolonged storage.
Sprouted barley	Contains beta-glucans and increases metabolism.	Enhances the prebiotic effect of inulin.	It can affect digestion due to its dietary fiber and active enzymes.
Liquorice root	Anti-inflammatory and antioxidant effects.	Improves the taste (sweet), can mask the bitterness of cereals	Glycyrrhizic acid can trap sodium in the body.

**Table 7:** Four dietary supplement recipes.

	Ingredient Quantity (g)				Inulin Content
	Jerusalem Artichoke	Sprouted Oats	Sprouted Barley	Liquorice Root	
Recipe 1	70	5	5	20	11.25%
Recipe 2	70	10	10	10	12.00%
Recipe 3	50	25	20	5	11.00%
Recipe 4	60	12.5	12.5	15	10.88%

**Table 8:** Organoleptic parameters of various dietary supplements.

	Appearance	Color	Smell	Taste	Consistency	Humidity
Recipe 1	coarse powder	beige-gray	strong, vegetative	sweet, with an unpleasant aftertaste	slightly lumpy	7.2%
Recipe 2	slightly heterogeneous	light beige	vegetative	tart	medium	6.9%
Recipe 3	heterogeneous	gray	sharp	bittersweet	lumpy	7.4%
Recipe 4	homogeneous	beige	pleasant, herbal	moderately sweet	loose	6.5%

**Table 9:** Water holding capacity, densities, and flowability for the four dietary supplement compositions.

	Water Holding Capacity (g/g)	Bulk Density (g/mL)	Compacted Density (g/mL)	Slope Angle (°)	Flowability
Recipe 1	1.022	0.50	0.62	42	mediocre
Recipe 2	0.316	0.58	0.72	35	good
Recipe 3	0.205	0.53	0.67	46	bad
Recipe 4	0.701	0.60	0.74	34	good

**Table 10:** Correlation analysis between dietary supplement components and inulin content.

	Jerusalem Artichoke	Sprouted Oats	Sprouted Barley	Liquorice Root	Inulin Content
**Jerusalem artichoke**	1	−0.946	−0.940	0.674	0.670
**Sprouted oats**	−0.946	1	0.989 *	−0.872	−0.396
**Sprouted barley**	−0.940	0.989 *	1	−0.878	−0.389
**Liquorice root**	0.674	−0.872	−0.878	1	−0.095
**Inulin content**	0.670	−0.396	−0.389	−0.095	1

* Marked correlations are significant at *p* < 0.05. *N* = 4 (casewise deletion of missing data).

**Table 11:** Physico-chemical and qualitative parameters of dietary supplement recipes.

	Recipe 1	Recipe 2	Recipe 3	Recipe 4
*Physico-chemical parameters*				
Mass fraction of protein (%)	11.42 ± 0.13	11.24 ± 0.10	11.70 ± 0.15	11.97 ± 017
Mass fraction of moisture (%)	7.06 ± 0.06	7.18 ± 0.08	7.49 ± 0.08	7.16 ± 0.05
Mass fraction of ash (%)	5.01 ± 0.04	5.46 ± 0.05	3.99 ± 0.02	4.30 ± 0.03
Mass fraction of fiber (%)	10.43 ± 0.12	7.39 ± 0.11	5.04 ± 0.08	9.24 ± 0.13
Titrated acidity (°T)	12.0 °T	12.0 °T	7.0 °T	9.0 °T
*Vitamin content* (*mg/100 g*)				
Vitamin E	25.34	52.45	32.78	57.75
Vitamin B1	0.12 ± 0.024	0.10 ± 0.020	0.13 ± 0.026	0.09 ± 0.018
Vitamin B2	0.15 ± 0.063	0.11 ± 0.046	0.16 ± 0.067	0.13 ± 0.055
Vitamin B3	0.64 ± 0.12	0.45 ± 0.09	0.68 ± 0.13	0.48 ± 0.10
Vitamin B5	0.53 ± 0.10	0.36 ± 0.06	0.59 ± 0.10	0.39 ± 0.07
Vitamin B6	0.17 ± 0.034	0.13 ± 0.026	0.20 ± 0.040	0.10 ± 0.020
Vitamin C	0.022 ± 0.0003	0.015 ± 0.0001	0.017 ± 0.0002	0.018 ± 0.0003
*Mineral content* (*mg/100 g*)				
Iron	1.25 ± 0.019	1.05 ± 0.015	1.20 ± 0.016	1.15 ± 0.012
Magnesium	32.20 ± 0.48	29.21 ± 0.35	30.34 ± 0.45	31.25 ± 0.43
Calcium	105.50 ± 1.09	110.63 ± 1.43	112.24 ± 1.68	98.38 ± 1.15
Potassium	325.30 ± 4.88	310.48 ± 4.22	320.63 ± 3.64	318.74 ± 3.96
Phosphorus	64.5 ± 0.84	59.28 ± 0.79	60.49 ± 0.80	62.03 ± 0.75
Iodine	Not detected			

**Table 12:** Amino acid content of dietary supplement samples.

	Recipe 1 (%)	Recipe 2 (%)	Recipe 3 (%)	Recipe 4 (%)
Arginine	2.388 ± 0.955	2.207 ± 0.883	1.249 ± 0.499	1.806 ± 0.723
Lysine	1.298 ± 0.441	0.331 ± 0.113	0.568 ± 0.193	0.365 ± 0.124
Tyrosine	0.649 ± 0.195	0.435 ± 0.130	0416 ± 0.125	0.384 ± 0.115
Phenylalanine	0.597 ± 0.179	0.635 ± 0.191	0.832 ± 0.250	0.846 ± 0.254
Histidine	0.088 ± 0.044	0.097 ± 0.048	0.061 ± 0.030	0.127 ± 0.063
Leucine + isoleucine	1.038 ± 0.270	0.836 ± 0.217	1.135 ± 0.295	0.922 ± 0.240
Methionine	0.519 ± 0.176	0.368 ± 0.125	0.363 ± 0.123	0.423 ± 0.144
Valine	0.882 ± 0.353	0.635 ± 0.254	0.870 ± 0.348	0.730 ± 0.292
Proline	0.623 ± 0.162	2.240 ± 0.582	1.967 ± 0.512	2.460 ± 0.640
Threonine	0.701 ± 0.280	0.535 ± 0.214	0.681 ± 0.272	0.615 ± 0.246
Serine	0.649 ± 0.169	0.702 ± 0.183	1.022 ± 0.266	0.884 ± 0.230
Alanine	0.934 ± 0.243	0.468 ± 0.122	0.757 ± 0.197	0.576 ± 0.150
Glycine	0.597 ± 0.203	0.535 ± 0.182	0.605 ± 0.206	0.538 ± 0.183

**Table 13:** Microbiological parameters for the four recipes.

	Recipe 1	Recipe 2	Recipe 3	Recipe 4
TMAFAM (CFU/g)	7.3 × 10^4^	7 × 10^4^	8.3 × 10^4^	6.3 × 10^4^
EC Group Bacteria (coliforms) in 1.0 cm^3^ of product	Not detected			
Yeast (CFU/g)	19	12	16	13
Mold growth rate (CFU/g)	39	14	10	8

Note: TMAFAM—Total Mesophilic Aerobic and Facultative Anaerobic Microorganisms; EC Group Bacteria—Bacteria of the Escherichia coli Group.

## Data Availability

The original contributions presented in the study are included in the article; further inquiries can be directed to the corresponding author.
